# Zeolites in Phenol Removal in the Presence of Cu(II) Ions—Comparison of Sorption Properties after Chitosan Modification

**DOI:** 10.3390/ma13030643

**Published:** 2020-02-01

**Authors:** Lidia Bandura, Małgorzata Franus, Jarosław Madej, Dorota Kołodyńska, Zbigniew Hubicki

**Affiliations:** 1 Faculty of Civil Engineering and Architecture, Lublin University of Technology, Nadbystrzycka 40, 20-618 Lublin, Poland; j.madej@pollub.pl; 2 Faculty of Chemistry, Maria Curie Skłodowska University, Maria Curie Skłodowska Sq. 2, 20-031 Lublin, Poland; d.kolodynska@poczta.umcs.lublin.pl (D.K.); z.hubicki@poczta.umcs.lublin.pl (Z.H.)

**Keywords:** fly ash, zeolite, chitosan, phenol, heavy metal ions

## Abstract

Nowadays, the contamination of water with phenol is a serious environmental problem. This compound occurs very often with heavy metal ions which makes purification of water even more difficult. This article presents the problem of the removal of phenol from aqueous solutions in the presence of Cu(II) ions on synthetic zeolite NaP1 and zeolite NaP1 modified with chitosan. The adsorbents were determined with the use of Fourier transform infrared spectroscopy (FT-IR), nitrogen adsorption/desorption isotherm, and scanning electron microscopy (SEM). The studies on isotherms and batch kinetics under diversified experimental conditions with respect to initial concentration, contact time, and pH were discussed. Both Cu(II) and phenol adsorption increases with the initial concentration. Different isotherm models correspond well with the data acquired through experiments. The kinetics of adsorption follows the pseudo-second order rate equation. The studies indicate that the obtained sorbents can be employed for efficient removal of phenol from wastewater in the presence of Cu(II) ions.

## 1. Introduction

There are many sources of phenol in the environment. This large group of compounds is of natural and anthropogenic origin. Natural sources of phenol and its derivatives are indirect products of natural decay of organic matter, products of forest fires, and products of metabolic processes occurring in living organisms and plants. The anthropogenic origin of phenols is associated with petroleum, petrochemical, paint, pesticide, plastics, pharmaceutical polymeric resin, leather, steel industries, and coal conversion. Phenol is an aromatic compound that was first isolated from coal tar in 1834 by Runge [1,2,3]. At room temperature and pressure, it is a white, hygroscopic crystalline solid. It becomes pink after exposure to light and air due to partial oxidation. Phenol is not completely soluble in water and behaves like a weak acid. The presence of phenol in water—even at low concentrations—can cause damage to the kidneys, pancreas, and liver, and can lead to paralysis of the central nervous system, protein degeneration, and tissue erosion [1]. Phenol at a concentration greater than 2 mg/L is toxic for fish and between 10–100 mg/L can cause death in most aquatic species [4]. Therefore, the US Environmental Protection Agency (EPA) introduced restrictions on the maximum phenol content (0.5 mg/L) in wastewater [5,6]. Phenol can persist in water causing different reactions, like chlorination, methylation, or nitration. The products of these reactions are more harmful for humans and the environment than phenol by itself.

Phenol is often found alongside such heavy metals as Cu(II) or Cd(II), which makes wastewater treatment even more complicated [7]. Copper is a trace element that regulates many body processes. Its minimum daily intake is 0.5 mg/L. However, when its concentration is lower or higher than required, natural processes of organisms are at risk. The US EPA announced that the maximum level of copper in drinking water is 1.3 mg/L. At higher concentrations, copper poisoning may lead to hypotension, jaundice, coma, hemostasis, and problems with the digestive system. Prolonged exposure to copper may also damage the kidneys and liver [8].

Despite the fact that various impurities exist in the natural environment, most of the papers related to removal of contaminants from water focus only on the one-component system of phenol or copper [9,10,11,12]. 

Many different methods of purification of wastewaters from phenol and copper ions were proposed, i.e., membrane processes, solvent extraction, ion exchange, adsorption, reverse osmosis, precipitation, distillation, advanced oxidation, electrochemical oxidation, and biological degradation [6].

The most effective method is adsorption because of the low initial cost, easily available adsorbents, simple operation, and high effectiveness. Adsorption techniques are used for the removal of toxic substances, such as heavy metals, phosphates, dyes, phenols, and organic compounds. Products, such as natural and synthetic zeolites [13,14], agricultural origin byproducts [15], clay minerals [16], and industrial wastes [17], are used for this purpose by many researchers due to their low cost and good capabilities for adsorption processes. As mineral materials, zeolites are very popular and have numerous environmental and industrial applications [18,19,20,21,22]. What is important, the recent studies showed they can be obtained from industrial wastes, such as fly ashes [23]. This method helps to reduce fly ash deposits. Due to the unique properties of zeolites, including ion exchange and sorption abilities, high specific surface area, and molecular-sieving behavior, they started to be used as adsorbents for the purification of contaminated water [24]. Among synthetic zeolites, NaP1 characterized by a pore diameter of 0.54 nm was reported as useful and valuable applicant for the water vapor adsorption and separation of many molecules [25,26,27,28]. In order to enhance the sorption abilities towards organic pollutants, zeolites can be modified with the use of organic modifiers [29]. Dong et al. reported adsorption of bisphenol A by zeolite modified with hexadecyltrimethylammonium bromide (HDTMA) [30]. The modified material exhibited greater retention of bisphenol A than unmodified zeolite due to electrostatic interactions of positively-charged heads of HDTMA with dissociated hydroxyls of bisphenol A, coordination of the oxygen atoms of bisphenol A by HDTMA positive charges, and the adsorption of uncharged bisphenol A by hydrophobic partitioning into HDTMA layers. Similar zeolite modifications were performed by Wołowiec et al. [31]. They used synthetic NaX zeolite and natural clinoptilolite modified with quaternary ammonium salts to the removal of volatile organic compounds (VOCs) and polycyclic aromatic hydrocarbons (PAHs) from aqueous solutions. Nevertheless, using surfactant-modified zeolites can be hazardous for environment to some extent. Therefore, it is important to find other organic compound which will be non-toxic and suitable for zeolite surface modification. One of the potential compounds is chitosan. Chitosan (poly(d-glucosamine)) has already been proven as a natural polymer suitable for the removal of metal ions, phenol, and phenol derivatives through chelation involving the amino and hydroxyl groups in the glucosamine unit [32]. 

This paper shall present for the first time the simultaneous removal of Cu(II) ions and phenol on zeolite NaP1 obtained from fly ash and NaP1 modified with chitosan (NaP1CS). Various experimental conditions, such as contact time, initial Cu(II) ions and phenol concentration, pH, and the effect of foreign ions, were investigated to establish the optimal conditions for Cu(II) as well as phenol removal from different waters and wastewaters. Moreover, thermodynamic and desorption studies were carried out and morphological properties of the materials were determined. The obtained results enabled establishing the conditions of production and application of the novel adsorbent. The proposed adsorbent is environmentally friendly, safe, and non-toxic.

## 2. Materials and Methods

### 2.1. Samples Preparation

The synthetic zeolite NaP1 was synthesized through the hydrothermal reactions of fly ash with NaOH [33]. The fly ash for zeolite synthesis was obtained from Kozienice Power Plant (Kozienice, Poland).. Its chemical composition determined with the use of ED-XRF (Epsilon 3x, Panalytical, Almelo, The Netherlands) revealed the presence of Na (0.18%), Al (21.52%), Si (47.4%), K (5.65%), Fe (14.71%), Cu (0.06%), Mg (0.8%), and Ca (2.96%). In order to modify the zeolite (NaP1), it was suspended in 200 mL of chitosan solution in glycolic acid and stirred for 32 h. Then, the suspension was adjusted to pH 9 using NaOH and washed with distilled water. The separation was carried out by filtration. After drying and grinding, the obtained material (NaP1CS) was ready for sorption experiments. 

### 2.2. Characterization of the Adsorbents

The mineral phases composition of NaP1 and NaP1CS was determined by the X-ray diffraction (XRD) method using a X’pert MPD X-ray diffractometer (Panalytical, Almelo, The Netherlands) with a goniometer PW 3020, Cu lamp and a graphite monochromator. Diffraction patterns were recorded by step scanning from 5 to 65, with a step size of 0.02°. HighScore Pro software version 4.1 (Panalytical, Almelo, The Netherlands) was used to process diffraction data. The identification of mineral phases was based on the PCPDFWIN ver. 1.30 formalized by JCPDS-ICDD (ICDD, Newtown, CT, USA).

Thermogravimetric (TG) analysis were performed using Netzsch STA 449F3 Jupiter apparatus (Netzsch, Selb, Germany). An air-dried sample was heated from 20 to 1000 °C, at the heating rate of 10 °C/min in flowing synthetic air.

Fourier transform infrared (FT-IR) spectra of the samples before and after sorption of phenol and Cu(II) ions were obtained using a FTIR spectrometer Cary 630 (Agilent Technologies, Santa Clara, CA, USA). All spectra were obtained at room temperature over the range of 4000–700 cm^−1^. 

The surface area, mean pore diameters and pore size distribution were determined by means of a Micromeritics 2040 automatic analyzer comprising the pressure transducer (Micromeritics, Norcross, GA, USA). The samples of zeolite were outgassed at 333 K for 24 h on the degas port of the analyzer. The sorption isotherms were created by adding nitrogen onto the sorbent at 77 K. 

The morphology of sorbents was determined by means of scanning electron microscopy, and surface elements were determined using energy-dispersive X-ray spectra (EDX) analysis SEM (LEO Electron Microscopy Ltd, Cambridge, UK, Model 1430).

### 2.3. Adsorption Experiments

The adsorption studies were conducted at room temperature and atmospheric pressure under batch conditions. It was carried out by introducing 0.1 g of NaP1 or NaP1CS into the 100 mL Erlenmeyer flask containing 20 mL of phenol and/or Cu(II) ions solution in the concentration range from 25 to 100 mg/L of each component. After shaking for 1–1440 min on a mechanical shaker manufactured by Elpin Plus (Lubawa, Poland), at 7 amplitude, 180 rpm, the zeolites were separated by filtration, and the concentration of phenol was measured using a UV–VIS spectrophotometer Cary 60 (Agilent Technologies, Santa Clara, CA, USA) by measuring absorbance at λ_max_ = 506 nm. The concentrations of Cu(II) in solutions were determined by means of the Spectr AA 240 FS atomic absorption spectrometer (Varian, Palo Alto, CA, USA). Cu(II) was detected at 324.7 nm. The device comprised a deuterium as well as a cathode lamp used for the Cu determinations. As a comparison, the titration method with the measurement-analytical set (Metrohm) was applied.

The investigation was carried out with chitosan from Sigma Aldrich (with the deacetylation degree of flakes > 75%). The solution of Cu(II) ions was prepared with the use of CuCl_2_·2H_2_O (POCH S.A., Gliwice, Poland). The phenol solution was prepared using phenol (POCH S.A., Gliwice, Poland).

The following formula was used to calculate the uptake of phenol and Cu(II) ions (mg/g):

*q*_t_ = (*C*_0_ − *C_t_*) *V*/*m*, (1)

The sorption percentage %S (%):

*S*(%) = (*C*_0_ − *C_t_*)/*C*_0_) × 100%, (2)

where *C*_0_ is initial solution concentration (mg/L), *C_t_* is the solution concentration after specific time *t* (min), *V* is the volume of the solution (L), and *m* is the dry weight of the adsorbent (g).

To investigate the effect of initial pH value on the removal of phenol in the presence of Cu ions, different solution pH values (4, 5, 6, 7, and 8) were adjusted by 0.1 M HCl and NaOH solutions. The pH values of the solutions were measured with the pH meter pHM82 (Radiometer, Copenhagen, Denmark).

In order to investigate the influence of phenol and/or Cu(II) concentrations, the single and binary systems of adsorption were employed. 

The effect of interfering of ions on phenol and the Cu(II) adsorption was determined in the presence of chloride (Cl^−^), sulfate(VI) (SO_4_^2−^), and nitrate(V) (NO^3−^) ions. The initial concentrations of interfering ions solution range from 100 to 500 mg/L, whereas the initial concentrations of phenol and Cu(II) were 100 mg/L each. This effect was investigated based on 0.02 L solution and 0.1 g NaP1 or NaP1CS placed in a mechanical shaker and shaken for 120 min at room temperature, at amplitude 7, and 180 rpm. The Vario 940 Professional IC (Metrohm, Herisau, Switzerland) ion chromatography method was used to measure the concentration of Cl^−^, SO_4_^2−^, and NO^3−^ ions.

### 2.4. Adsorption Isotherms

Sorptive properties of zeolites (NaP1, NaP1CS) were measured through the analysis of the phenol sorption isotherms. The analysis of equilibrium isotherm data was conducted with the use of the Langmuir, Freundlich, Dubinin–Radushkevich, and Temkin models at 293, 313, and 333 K.

The Langmuir isotherm model indicates that the adsorbent surface is characterized by a certain number of sites. The adsorbate molecules can be adsorbed until the capacity of the adsorbent is finite. The linear form of this isotherm model can be represented as follows:

*C_e_*/*q_e_* = 1/*bq*_0_ + 1/*q*_0_, (3)

where *q*_0_ (mg/g) is the maximum adsorption capacity, *q_e_* (mg/g) is the amount of adsorbed Cu(II) ions or phenol per of adsorbent mass, *C_e_* (mg/L) is the unabsorbed Cu(II) ions or phenol concentration in the solution, and *b* is the Langmuir adsorption constant (L/mg) [34,35].

The Freundlich isotherm is the empirical equation used for description of heterogeneous systems and can be applied in the multilayer adsorption. The linear form of the Freundlich isotherm can be expressed by the following Equation (4):

log*q_e_* = log*K_F_* + (1/n log*C_e_*), (4)

where *K_F_* ((mg/g(L/mg)^1/n^) and *n* are the Freundlich constants [36].

The Dubinin–Radushkievich isotherm is generally applied to find the adsorption mechanism onto the heterogeneous surface. The linear form of the Dubinin–Radushkievich isotherm can be expressed by Equation (5):

log*q_e_* = ln*q_D_* − *K_DR_*·ε^2^, (5)

where *q_D_* (mg/g) denotes the theoretical isotherm saturation capacity, ε corresponds to the Polanyi potential, whereas *K_DR_* (mol^2^/J^2^) is the constant pertaining to the mean free energy of adsorption per mole of the adsorbate. The equilibrium linked to the Polanyi potential can be expressed as follows:

ε = *RT* ln(1 *+* 1/*C_e_*), (6)

where *R* (8.314 J/mol K) is the gas constant, *T* (K) is the temperature, *C_e_* (mg/L) is the concentration at equilibrium [37]. 

The Temkin adsorption isotherm assumes that adsorption heat decreases linearly as a result of the interactions between the adsorbent and the adsorbate. The linear form of this isotherm model can be represented as follows:

*q_e_* = *B* ln*k_T_* + *B* ln*C_e_*, (7)

where *B* equal to *RT*/*b_T_* (J/mol) is related to the adsorption heat, *b_T_* is Temkin constant connected to the adsorption heat, *R* (8.314 J mol K) is the gas constant in temperature *T* (K), *k_T_* (L/g) is the Temkin equilibrium constant related to the maximum binding energy [37].

### 2.5. Adsorption Kinetics

The experimental data were described using the intraparticle diffusion, pseudo-first order, pseudo-second order, and Elovich kinetic models. 

Langergren and Svenska proposed the pseudo-first order model. The linear form of this model can be expressed by the following Equation (8):

ln(*q_e_* − *q_t_*) = ln*q_e_* − *k*_1_*t*, (8)

where *q_e_* (mg/g) denotes the amount of Cu(II) or phenol adsorbed at equilibrium, *q_t_* (mg/g) is the amount of Cu(II) or phenol adsorbed at any time, and *k*_1_ (1/min) is the rate constant [38].

The pseudo-second order kinetic model can be expressed by linear equation Equation (9):

t/*q_t_* = 1/(*k_2_q_e_*^2^) + *t*/*q_e_*, (9)

where *k*_2_ (g/mg h) is the rate constant [38,39].

The intraparticle diffusion model is employed for determining the intraparticle diffusion mechanism. This empirical formula is shown in Equation (10):

*q_t_* = *k_i_ t*^1/2^ + *C*, (10)

where *k_i_* (mg/g min^1/2^) is the rate constant, and *C* (mg/g) is the diffusion constant [40].

Generally, the linear equation of the Elovich model is expressed as follows:

*q_t_* = 1/*β* ln(*αβ*) + 1/*β* ln*t*, (11)

where: α is the constant related to the initial adsorption rate (mg/g min), while *β* corresponds to the desorption constant (g/mg). Both constants can be obtained from the intercept and slope of the plot of *q_t_* versus ln*t* [41].

### 2.6. Thermodynamic Study

The feasibility and nature characterizing the adsorption process were estimated using such thermodynamic parameters as the standard Gibbs free energy (ΔG°), the standard enthalpy (ΔH°), as well as the standard entropy (ΔS°) change. These parameters were calculated with the use of the following equations:

ΔG° = −*RT* ln*K_L_*, (12)

ln*K_L_* = − (ΔH°/*RT*) + ΔS°/R, (13)

where K_L_ (L/mg) corresponds to the Langmuir constant, R (8.314 J/mol K) [42].

## 3. Results and Discussion

### 3.1. Characteristics of Adsorbents

**Figure 1:**
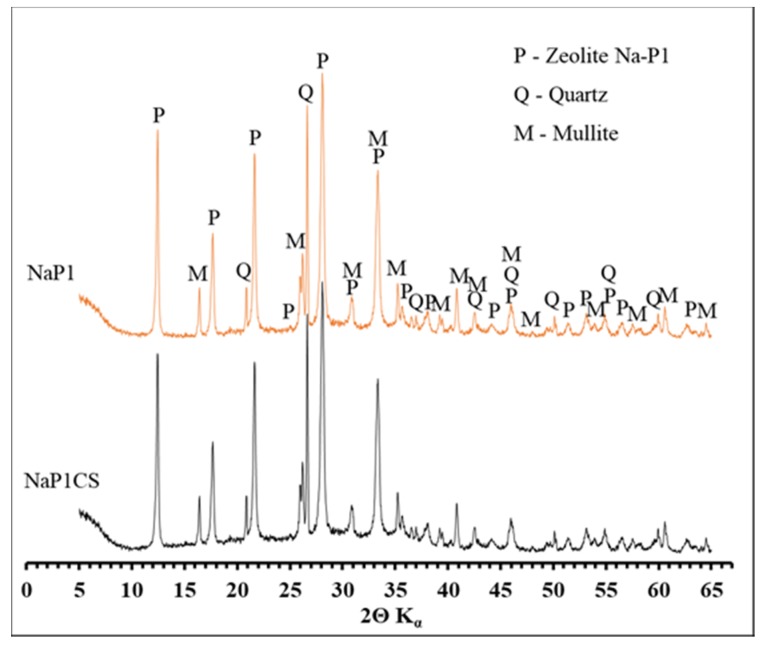
XRD patterns of NaP1 and NaP1CS.

Figure 1 shows the XRD patterns of the NaP1 zeolite and the NaP1CS. For NaP1, the diffraction peaks attributed to zeolite phase (d_hkl_ = 2.68; 3.17; 4.10; 5.02; 7.10 Å), mullite (d_hkl_ = 1.52; 2.54; 3.40; 3.42; 5.40 Å), and quartz (d_hkl_ = 1.37; 1.54; 1.81; 3.34; 4.26 Å) were recognized. The diffractogram of NaP1CS exhibits almost the same patterns as NaP1. The main difference is the slightly higher background line level between 10 and 35° 2 theta attributed to the presence of amorphous chitosan.

**Figure 2:**
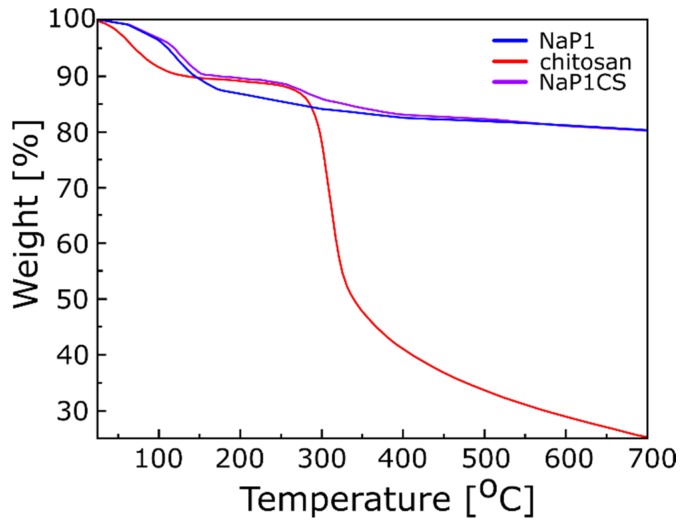
TG thermograms of chitosan, NaP1, and NaP1CS.

The TGA thermograms of NaP1, chitosan, and NaP1CS samples are shown in Figure 2. TGA thermogram of chitosan indicates the two weight losses: one from 40 to approximately 120 °C due to evaporation of water, and second from 300 °C up to 450 °C due to the degradation of the saccharide rings. TG curve of NaP1 reveals water loss stage in the temperature range between 100 and 200 °C (which constitutes about 15% of the sample weight). Then, the sample remains quite stable until the end of measurement. However, the TGA of NaP1CS resembles both the TGA of chitosan and NaP1. In the first stage of heating (temperature up to 150 °C), the TGA curve is similar to the NaP1. Then, in the temperature range of 150–300 °C, it almost coincides with the chitosan TGA curve (Figure 2). Above 300 °C small degradation of the saccharide rings can be observed. In the last stage of heating, the curves for NaP1 and NaP1CS are almost identical that indicates the thermal degradation of chitosan. TGA analysis confirms that the modification of NaP1 by chitosan was successful. 

**Figure 3:**
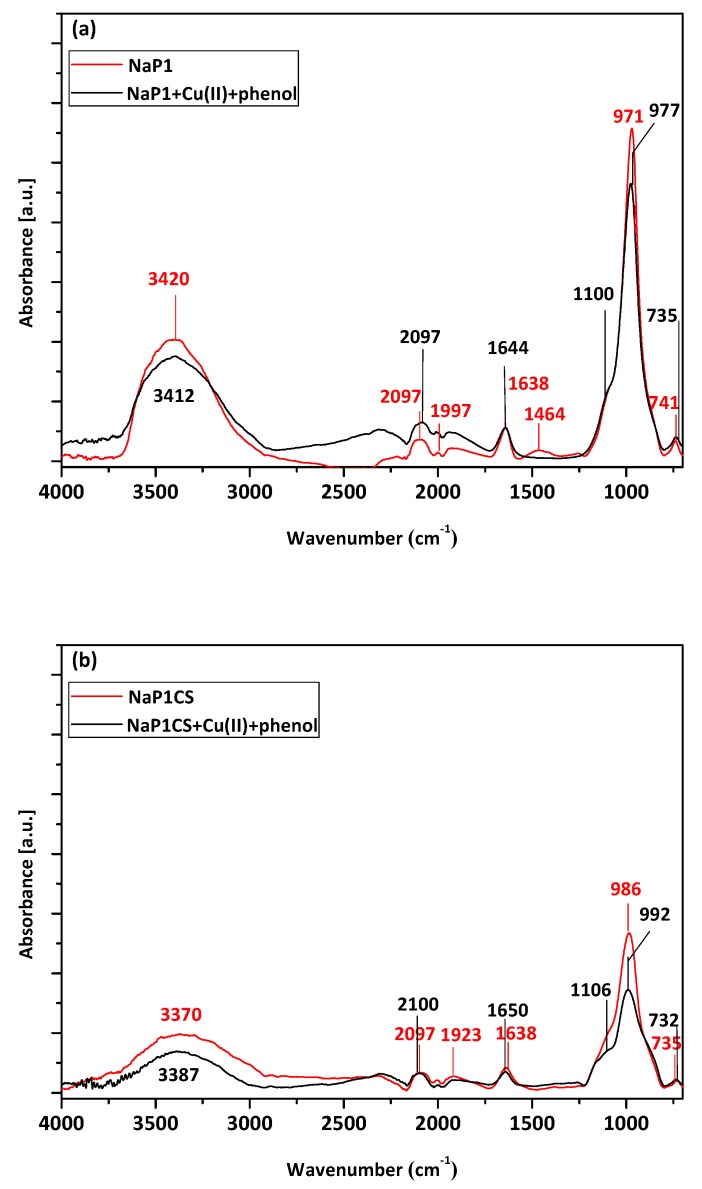
ATR-FTIR spectra of (**a**) raw NaP1 and NaP1 after sorption of Cu(II) ions and phenol, and (**b**) raw NaP1CS and NaP1CS after sorption of Cu(II) ions and phenol.

The FT-IR analysis (Figure 3) was performed on NaP1 and NaP1CS zeolites before and after the sorption of Cu(II) and phenol. The spectrum of NaP1 reveals typical bands of zeolite of gismondine type. The band located at 1638 cm^−1^ corresponds to the bending vibration of water molecules and is attributed to the presence of zeolitic water. A broad bend with maximum at 3420 cm^−1^ is associated with stretching vibrations of O–H bonds present in water molecules adsorbed on the surface of the material. A strong bands with the maximum at about 977 cm^−1^ corresponds to asymmetric stretching vibrations occurring in the Si–O–Si(Al) bridges of the zeolitic skeleton. Bands present in the wavelength range of 400–800 cm^−1^ can be attributed to the so-called ‘pseudolattice range’ which reflects vibrations of SBU units of zeolites and also to the external vibration of zeolitic water. Based on the literature, chitosan exhibits the typical band in the region of 3300–3500 cm^−1^ associated with the stretching vibrations of the –OH and N–H bonds present in hydroxyl and amino groups, respectively. It also reveals the band in the region of 1600–1650 cm^−1^ which corresponds to the N-H bond of the amino group, and a band at 1000–1100 cm^−1^ corresponding to C–O–C group. The FTIR spectra of NAP1CS constitute the combination of NaP1 and chitosan with strongly visible zeolitic peaks and bands related to the presence of chitosan. The peak attributed to zeolitic water is located atthe wavelength of 1630 cm^−1^ (stronger than for NaP1 before modification) and a visible band at 1100–1200 attributed to C–O–C groups related to chitosan. After Cu(II) and phenol adsorption, no significant changes can be observed in FTIR spectra. Maxima of the characteristic bands have not moved. Nevertheless, the intensity of peaks changes slightly and a band with maximum at a wavelength 1000 cm^−1^ is less intensive after adsorption for NaP1, and the opposite trend can be observed for NaP1CS after adsorption. The initial changes in the FTIR spectra caused by the heavy metal ion sorption should be observed in the range of the pseudolattice vibrations, i.e., 700–500 cm^−1^. For example, the band at 674 cm^−1^ can be considered as an “indicator” with intensity changing with metal sorption [43,44]. 

**Figure 4:**
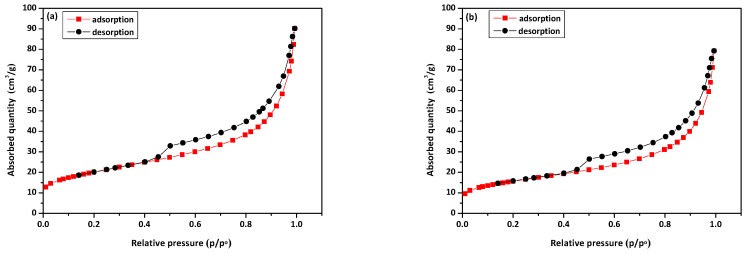
Nitrogen adsorption/desorption isotherms at 77K for (**a**) NaP1 and (**b**) NaP1CS after sorption of Cu(II) and phenol.

Figure 4a,b shows the nitrogen adsorption/desorption isotherms of NaP1 and NaP1CS after the sorption of Cu(II) and phenol. According to IUPAC classification type IV isotherms were observed on both NaP1 and NaP1CS. This type of isotherm is attributed to multilayer adsorption of physical nature on porous solids and is characteristic for mesoporous materials. For both materials, a hysteresis loop of H2/H3 type could be observed between 0.46 and 0.98 p/p_o,_ which suggests a presence of “ink-bottle” or slit shaped mesopores. Isotherms on NaP1 and NaP1CS have a similar shape but NaP1CS adsorbed less nitrogen. This indicates that chitosan modification caused partial pore blocking and reduction of the specific surface area. Table 1 presents textural parameters of examined adsorbents before and after phenol and Cu(II) adsorption. 

Before Cu(II) and phenol adsorption, specific surface area S_BET_ was higher for NaP1 than for NaP1CS. It can be assumed that after modification chitosan blocked some pores of NaP1 zeolite. The data obtained by the nitrogen adsorption/desorption experiments shows a decrease of the specific surface area for the sample NaP1 after the sorption of Cu(II) and phenol. Marakatti et al. reported that zeolite specific surface can decrease after some metal ions exchange [45]. However, for NaP1CS the specific surface area increased slightly after the sorption. The latter parameter, that is the total pore volume, was almost three times higher for NaP1 than NaP1CS. The sorption of Cu and phenol caused a reduction of these values for NaP1 and NaP1CS. Moreover, a decrease in BJH desorption average pore width (D_p_) was observed for the samples after sorption process. The total pore volume and average pore width decreased which means that after the sorption of Cu(II) and phenol the zeolite pores were partly blocked. 

The SEM analysis is widely employed for examining the characteristics of the surface and morphological features of the adsorbents. In this study, the SEM investigations were applied to study the surface changes after Cu(II) and phenol sorption. The SEM images show that the synthetic zeolite NaP1 was successfully modified by chitosan to obtain NaP1CS and the surface of zeolite changed after sorption.

**Figure 5:**
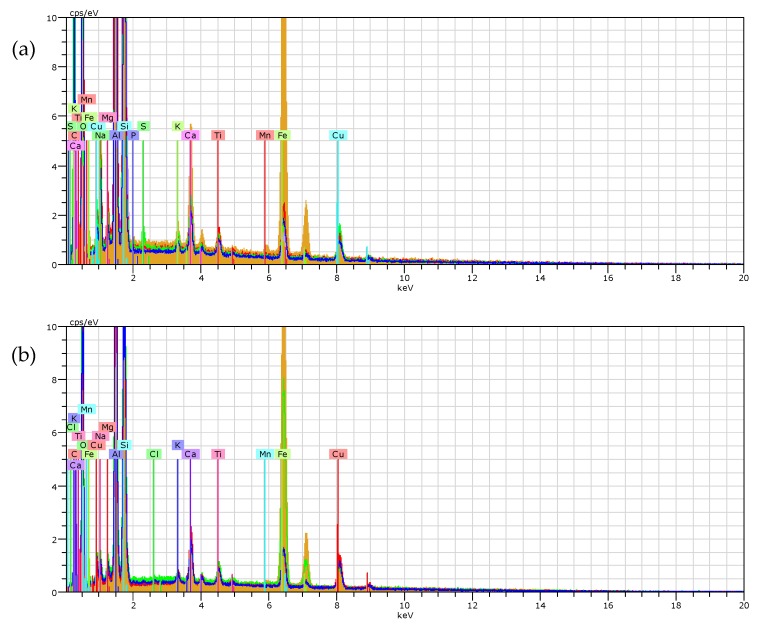

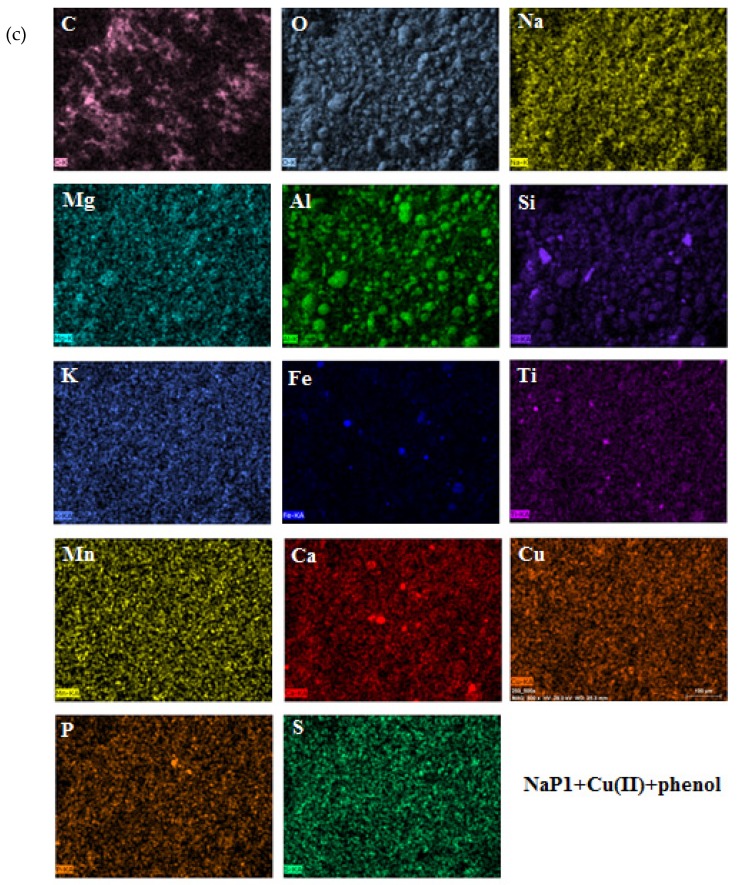

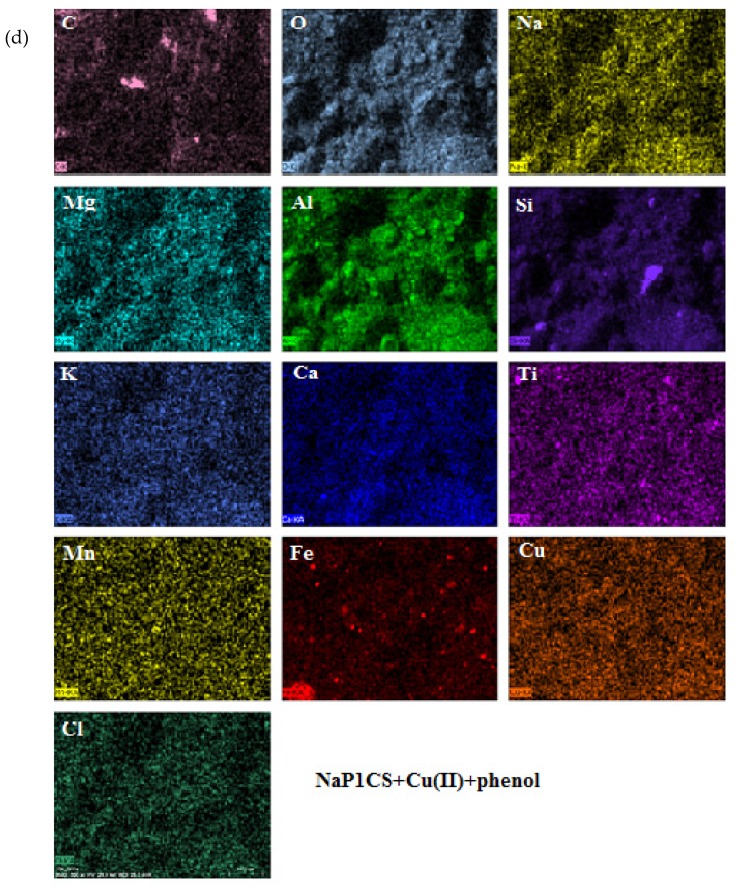
SEM–EDS analysis of (**a**) NaP1 and (**b**) NaP1CS after sorption of Cu(II) and phenol with the corresponding X-ray maps of C, O, Na, Mg, Al, Si, K, Fe, Ti, Mn, Cu, Ca, P, and S on (**c**) NaP1, and (**d**) NaP1CS (magnification: 1000×).

The SEM-EDX analysis was performed to confirm the occurrence of adsorbed Cu(II) ions. This method was used to find the location of components in zeolites. The data obtained after the sorption of Cu(II) and phenol and the distribution of elements through mapping is presented in Figure 5. The main elements of NaP1 are O 44.54%, Si 18.52%, Al 13.73%, and C 7.32%, similarly to NaP1CS they were O 36.37%, Si 19.03%, Al 18.59%, and C 3.49%. Before sorption on NaP1 and NaP1CS, the rate of Cu was 0.31% and 0.26%, respectively. After Cu(II) sorption, it was 1.98% and 3.14% for NaP1 and NaP1CS, respectively. This means that the modified zeolite NaP1CS proved to be a better sorbent than NaP1. The EDX spectra show the characteristic peaks of copper (Figure 5a,b).

### 3.2. Effect of Initial Concentration and pH Dependence

**Figure 6:**
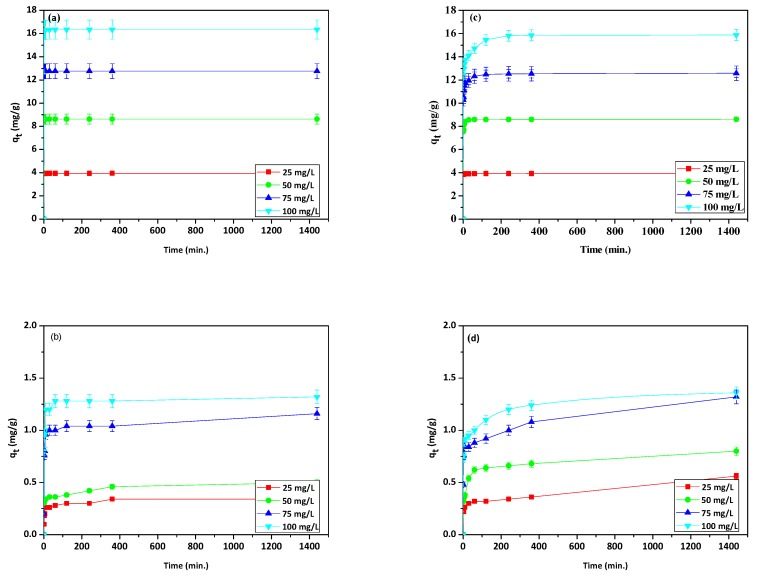
Effect of contact time and initial concentration on sorption capacity of (**a**) Cu(II), (**b**) phenol on NaP1, (**c**) Cu(II), and (**d**) phenol on NaP1CS (*C*_0_ = 25, 50, 75, 100 mg/L of Cu(II) and phenol, *m* = 0.1 g).

In order to examine the effect of initial concentrations of Cu(II) and phenol in the dual system, the following concentrations of each component were used: 25, 50, 75, and 100 mg/L. Figure 6a–d presents the graphs of adsorption capacity *q_t_* versus time. The obtained results show that the sorption capacity increased together with the contact time of the phase, as well as the initial concentrations of Cu(II) and phenol. A similar trend was observed for phenol adsorption by Ge et al. [46], Bahdod et al. [40], Abdelwahab and Amin [47], and Yousef [48]. Cu(II) maximal adsorption was similar for NaP1 and NaP1CS, whereas in the case of phenol adsorption, it was higher for NaP1CS at initial concentrations of 25 mg/L and 50 mg/L. It could be observed that Cu(II) adsorbed faster than phenol on both adsorbents. In general, the adsorption process was slower in the case of NaP1CS than in the case of NaP1 (by observing the trend to achieve the equilibrium state). The obtained results indicate that the kinetics and mechanism of adsorption is different on modified zeolite NaP1CS than on unmodified NaP1. 

**Figure 7:**
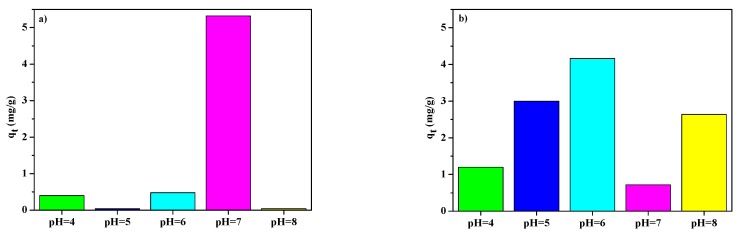
pH dependence of phenol sorption capacity on (**a**), NaP1 (**b**) NaP1CS.

In an aqueous solution, pH is an essential factor affecting the phenol adsorption process, the ionization degree of phenol as well as the surface charge of the adsorbent [9]. Figure 7a,b show the phenol adsorption capacities in the 4–8 pH range. 

For NaP1 one can see that the sorption capacity of phenol at pH values of 4 and 6 was similar, at pH 7 it significantly increased, and then, at pH 8, the rapid reduction in the sorption capacity occurred. However, for NaP1CS the sorption capacity increased up to pH 6 (maximum adsorption capacity) and decreased at pH 7 and pH 8. At pH the sorption capacity was similar to that at pH 5. Phenol has a level of hydrophilicity due to its hydroxyl group. As a result, phenol competes with water for adsorption sites in the aqueous phase. pK_a_ for phenol is 9.89 and at the pH value above pK_a_ phenol is in the dissociated form as (C_6_H_5_O^−^) phenolate ions, while the surface of zeolite is charged negatively. Therefore, the repulsion force between the surface of zeolite and C_6_H_5_O^−^ is observed. It can cause the decrease of sorption capacity [9,49]. However, the effect of the CS should be taken into account. The surface charge of the NaP1 and NaP1CS zeolite also varies with the pH of the aqueous solution. At low pH the protonation of the available zeolite surface and –OH and –NH_2_ groups of CS occurs. It may cause that sorption of phenol by hydrogen bonding can be hindered. As the pH rises, the hydroxyl and amino groups of NaP1CS surface becomes less protonated, which increases their ability of binding to positively charged Cu(II) and increases the formation of hydrogen bonding with the hydroxyl groups of phenol. Moreover, the amine group of CS can remove Cu(II) ions by ion exchange/chelating mechanism and by surface complexing. A gradual increase of pH leads to the formation of complex ions CuOH^+^ and precipitation to Cu(OH)_2_. This may enhance the formation of hydrogen bonding with the hydroxyl groups of phenol. The literature on the subject says that interactions between Cu(II) and phenol can also be observed in mixed Cu(II)-phenol solutions. The greatest phenol adsorption capacity was obtained at pH 7 for NaP1 and at pH 6 for NaP1CS. This difference can be explained by different surface charge on the studied materials at around neutral pH. Therefore, the pH value of 6 was chosen for further adsorption experiments.

### 3.3. Single and Dual Adsorption of Cu(II) and Phenol

**Figure 8:**
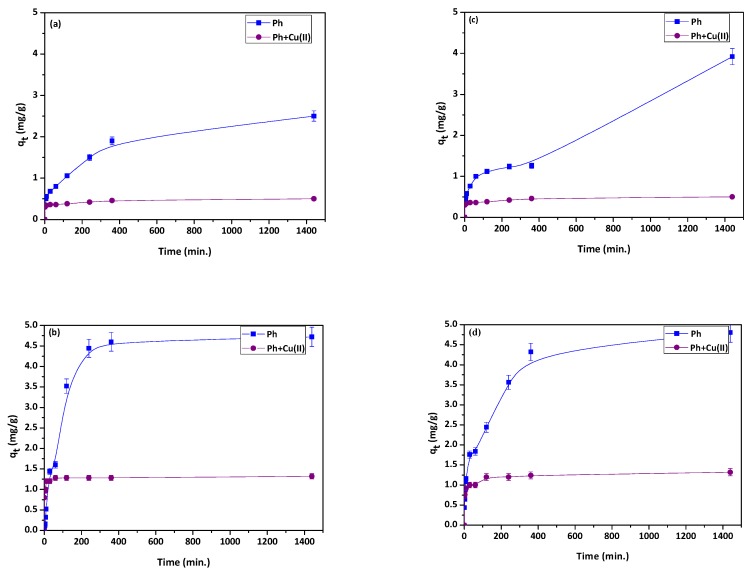
Effect of contact time for the single system of phenol and the dual one of Cu(II) ions and phenol on (**a**) NaP1 *C*_0_ = 50 mg/L; (**b**) NaP1 *C*_0_ = 100 mg/L; (**c**) NaP1CS *C*_0_ = 50 mg/L; (**d**) NaP1CS *C*_0_ = 100 mg/L), *m* = 0.1g.

The adsorption experiments for phenol sorption were performed in the single and dual systems of Cu(II) and phenol, and are presented in Figure 8a–d. 

The sorption percentage for phenol at the initial concentration 50 and 100 mg/L were 25% and 23.6% for NaP1 and 39.2% and 24% for NaP1CS for the single component solution. In the single component systems, the maximal sorption capacity in mg/g towards phenol was higher for zeolite modified with chitosan at both initial concentrations. For the dual sorption of phenol and Cu(II), the rates of sorption of phenol were lower than in the single system and they were 4.8% and 6.2% for NaP1 and 7.6% and 6.2% for NaP1CS, respectively for *C*_0_ = 50 and 100 mg/L. In the dual component systems, the maximal sorption capacity towards phenol was also higher for NaP1CS, but for lower initial concentration (as it was in the case of single component system (Figure 4)). For the concentration of 100 mg/L, sorption capacity towards phenol in dual component system was comparable for modified and unmodified zeolite.

During the adsorption in the poly-component systems, the adsorbate molecules compete with each other for active sites on the adsorbent surface. In the presented examinations, Cu(II) ions and phenol molecules adsorbed on NaP1 and NaP1CS simultaneously, but the mechanisms of adsorption were different for both compounds. 

The mechanism of Cu(II) ions removal by zeolites from fly ash has been thoroughly studied by Kyzioł-Komosińska et al. [14]. It can be stated that Cu(II) ions removal is the result of two phenomena: (i) bonding of Cu^2+^ cations by ion-exchange process (Cu^2+^ exchange Na^+^ cation in the zeolite framework) and (ii) precipitation of Cu(II) ions to Cu(OH)_2_. The mechanism of Cu(II) ions adsorption by chitosan/clay/magnetite composite was also studied by Cho et al. [50], and by chitosan–zeolite composites by Wan Ngah et al. [51]. Based on these researches, it can be assumed that Cu(II) ions bind to amine and hydroxyl groups in the chitosan through chelation and form stable complexes. It was proved that in chitosan-zeolite composite the following binding sites for Cu(II) ions occurred: –NH_2_, NH^3+^, –CH_2_OH (from chitosan), and SiO (from zeolite). Therefore, one can conclude that the Cu(II) ions adsorption process on NaP1CS is a result of chelation of the ions by chitosan functional groups and ion-exchange/adsorption in the available pores of zeolite matrix.

According to Yousef et al. [48], adsorption of phenol takes place mainly on the outer surface of zeolite rather than in the pores, and repulsion forces between phenol–phenol and phenol–adsorbent surfaces occur. Chaouati et al. [52] draws attention to the role of Si/Al ratio of zeolite in phenol adsorption. The greater the Si/Al ratio, the greater the hydrophobic character of the zeolite and the higher phenol uptake. Chitosan plays a significant role in phenol adsorption [1,53]. The hydroxyl groups in chitosan chain form hydrogen bond with –OH group present in phenol molecule.

Phenol removal from aqueous media has been studied extensively on different kind of adsorbents. Table 2 summarizes the values of adsorption capacities towards phenol in single-component system for different types of adsorbents. This study revealed that the sorption capacity of phenol was in the range of 4.5–5 mg/g in single-component system for the initial concentration 100 mg/L.

Studies related to the phenol removal in the presence of metal cations are very random. Wang et al. [56] examined the removal of phenol in the presence of Zn(II) ions on organomontmorillonites (OMt). Adsorption capacity of phenol in the dual system was in the range of 0.1–2 mg/g depending on the adsorbent type, whereas Zn(II) ions sorption remained on the level of 4 mg/g. Lalhmunsiama et al. [57] studied the removal of phenol in the presence of Hg(II) on functionalized activated carbon derived from areca nut waste with the result of sorption capacity for phenol in the range of 3.27–4 mg/g. Figure 6 shows that adsorption capacity of NaP1 and NaP1CS in dual component system reached about 1.25 mg/g for the initial concentration of 100 mg/L, and it was significantly higher than the adsorption capacity of NaP1 in the same experimental conditions. It may be concluded that among organo-mineral materials, NaP1CS could be considered as an efficient and environmentally friendly adsorbent for phenol.

### 3.4. Effect of Interfering Ions

**Figure 9:**
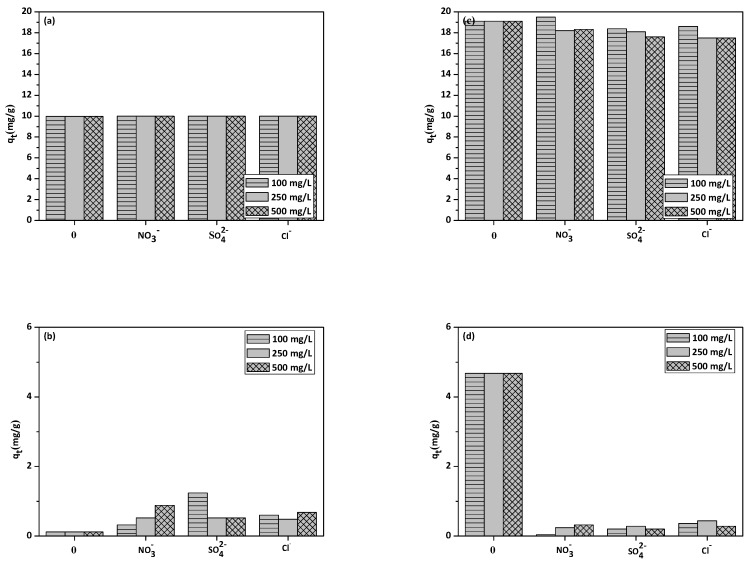
Effect of interfering ions on sorption capacity of (**a**) Cu(II), (**b**) phenol on NaP1, (**c**) Cu(II), and (**d**) phenol on NaP1CS (100, 250, 500 mg/L of NO_3_^−^, Cl^−^, SO_4_^2−^, *C*_0_ = 50 mg/L phenol–50 mg/L Cu(II), *m* = 0.1g, *t* = 120 min).

The influence of interfering ions (NO_3_^−^, SO_4_^2−^, Cl^−^) on Cu(II) and phenol adsorption on NaP1 and NaP1CS was investigated. The obtained results are shown in Figure 9a–d. They indicate that the addition of different amounts of interfering ions does not significantly affect the sorption capacity. 

For example, Cu(II) ions sorption capacity on NaP1 without the addition of interfering ions was 9.98 mg/L and with the addition of NO_3_^−^, SO_4_^2−^, Cl^−^ it was 9.99 mg/L for 100 mg/L. The most significant influence was observed for the phenol sorption on NaP1CS, where the sorption capacity decreased from 4.68 mg/L to 0.04 mg/L, 0.2 mg/L and 0.36 mg/L after the addition of 100 mg/L NO^3−^, SO_4_^2−^, Cl^−^, respectively.

### 3.5. Isotherm Models

The adsorption isotherm presents the distribution of adsorbate molecules between the solid and liquid phases in the adsorption process. Fitting the experimental data into the isotherm model and finding an appropriate model for the process is important for the purpose of modelling. Table 3 and Table 4 list the parameters obtained from the Langmuir, Freundlich, Temkin, and Dubinin–Radushkevich isotherm equations at 293, 313, and 333 K for the Cu(II) and phenol adsorption, respectively. 

The removal of Cu(II) on NaP1 and NaP1CS was not dependent on the temperature since the adsorption capacity remained at a similar level at 293, 313, and 333 K. On the other hand, the adsorption of phenol on NaP1 decreased with the increase of temperature. This indicates that the adsorption of phenol was exothermic process [48,49]. At high temperature, the thickness of the boundary layer decreases, due to the increased tendency of the metal ions to escape from the zeolite surface to the solution phase, which results in a decrease in adsorption as temperature increases. Similar results were achieved in the case of the bisphenol-A adsorption capacity of the Xiangjiang River sediments in China [58]. In that case, it was observed that, with an increase in temperature, there is a decrease in the value of bisphenol-A adsorption capacity. It is also known that the adsorption capacity of dyes can increase or decrease at higher temperatures depending on the nature of the reaction and other controlling factors [59]. Generally, if the reaction process is endothermic, adsorption can increase at higher temperature due to increased surface coverage, expansion, and creation of reactive and active sites. However, when the reaction is exothermic, a decrease in adsorption at higher temperature is observed. The Langmuir model is widely applied, which assumes that the adsorbate molecules can be adsorbed only on a specific number of sites of adsorbent surface. Only the monolayer adsorption without the interactions between the adsorbed molecules occurs. The energy of sorption for all sites is the same. The highest determination coefficient (*R*^2^) for the Langmuir model was obtained for Cu(II) ions on NaP1 and NaP1CS. Application of the Langmuir model are not useful for the description of sorption isotherms of phenol onto NaP1 and NaP1CS. *b* is the Langmuir adsorption constant related to the affinity for the binding sites. The values of *R_L_* show the isotherm types: unfavorable (*R_L_* > 1), linear (*R_L_* = 1), favorable (0 < *R_L_*< 1) or irreversible (*R_L_* = 0) [60]. Based on these assumptions, it can be concluded that the adsorption process for Cu(II) and phenol on NaP1 and NaP1CS is favorable. 

The Freundlich isotherm in its linearized form can be expressed by Equation (4). It assumes that the adsorption processes take place on a heterogeneous adsorbent surface, and in contrast to the Langmuir isotherm, it can be applied for multilayer adsorption. The Freundlich constants *K_F_* and *n* are obtained from the plot of log *q_e_* versus log*C_e_* and are related to the slope of 1/*n* and the intercept of log*K_F_*. The adsorption process tends to be favorable when the value of *n* is in the range of 1 to 10. Whereas, when the *n* value is higher, it implies strong interactions between the sorbent and Cu(II) ions or phenol [61]. As follows from Table 2 and Table 3, the *R*^2^ values were the highest for phenol on NaP1 and NaP1CS at each temperature, which indicates that the adsorption process can be described by the Freundlich isotherm. Similar results for phenol adsorption were obtained by Yousef et al. [48]. Besides, the values of *n* are in the range of 1 to 10, which indicates the favorable process.

The values of *q_D_* and constant *K_DR_* were calculated from the intercept and slope of the Dubinin–Radushkievich linear plots of ln*q_D_* versus *ε^2^*. The value of *K_DR_* provided the information about the mean free energy (*E*) of adsorption. This parameter helps to define the adsorption type. If the value is higher than 16 kJ/mol, it is chemisorption, in the case 8–16 kJ/mol, it is ion exchange, and for less than 8 kJ/mol, it is physical adsorption [62,63]. The research results indicate that for Cu(II) ions on NaP1 and NaP1CS, the values of *E* were 8.71–13.94 kJ/mol, which suggests that the adsorption is due to the exchange of ions. A different trend was observed for the phenol adsorption, which is a physical one, because the values of *E* ranged from 4.88 to 7.96 kJ/mol. Similar results were obtained by Chaouati et al. [52].

The Temkin isotherm indicates that the effects of some indirect adsorbate/adsorbate interactions in adsorption processes can take place. The constant *A* is related to the adsorption potential and *B* to the adsorption heat [64]. 

### 3.6. Kinetic Studies

To explain the Cu(II) ions and phenol adsorption on NaP1 and NaP1CS, the kinetic data are fitted by the pseudo-first, pseudo-second order, intraparticle diffusion, and Elovich models. The obtained kinetic parameters of the kinetic adsorption studies are shown in Table 5 and Table 6. One can see that the kinetics of Cu(II) ions and phenol removal by NaP1 and NaP1CS proved to be the pseudo-second-order model. The above statement is confirmed by the highest determination coefficient (R^2^) as well as by the fact that the theoretical *q_e_* could be compared with the experimental ones. For Cu(II) on NaP1 and NaP1CS, the *R*^2^ values of the pseudo-first order model are in the range of 0.550–0.994, for phenol on NaP1 and NaP1CS, they are of 0.564–0.918. On the other hand, the *R*^2^ values corresponding to the pseudo-second order model are greater than 0.999 for Cu(II) ions on NaP1 and NaP1CS, for phenol they are in the range from 0.974 to 0.999 on NaP1 and NaP1CS. With the increasing concentration, the sorption capacity for Cu(II) and phenol on NaP1 and NaP1CS also increased. The factors *k*_1_ and *k*_2_ slightly decreased with the increase of initial concentration on NaP1. For NaP1CS, the value of *k*_1_ raised to 50 mg/L and then dropped. The values of these parameters for phenol on NaP1 and NaP1CS do not exhibit downward or upward trends with the increasing initial concentration. That is, the reaction rate decreases with the increasing initial concentration of Cu(II). The higher initial concentration was, the longer period it took to achieve the equilibrium. 

Moreover, the experimental data were fitted into the intraparticle diffusion model and the obtained parameters are also presented in Table 4 and Table 5. 

**Figure 10:**
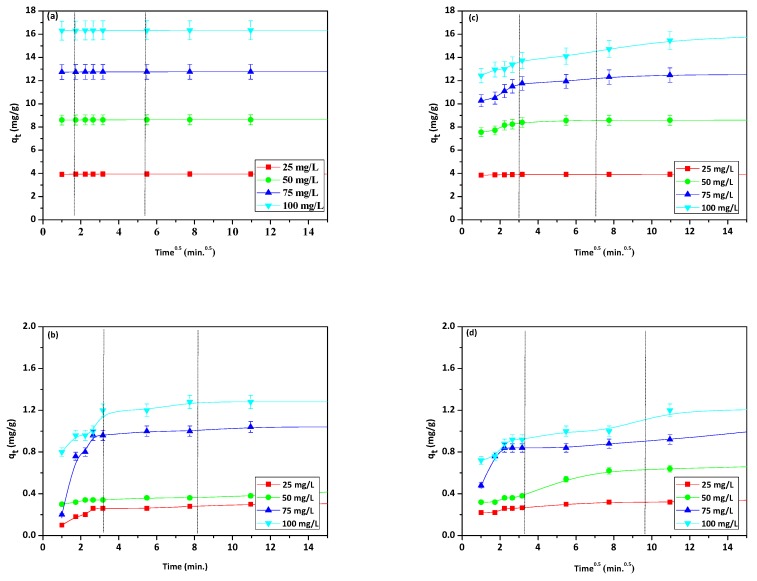
Kinetic plots of intraparticle diffusion model for sorption of (**a**) Cu(II), (**b**) phenol on NaP1, (**c**) Cu(II), and (**d**) phenol on NaP1CS (*C*_0_ = 25, 50, 75, 100 mg/L of Cu(II) and phenol, *m* = 0.1 g, *t* = 120 min).

Since the pseudo-first order and pseudo-second order kinetic models do not identify the adsorption diffusion mechanism, the intraparticle diffusion model can be tested to define the rate-controlling steps. In the intraparticle diffusion model, C (mg/g) is a constant providing information about the thickness of boundary layer, which can be calculated from the intercept and slope of the plots of *q_t_* vs. *t*^0.5^ [65]. According to the model, if this plot gives a straight line passing through the origin, the adsorption process is controlled by the intraparticle diffusion whereas, if the data exhibits multilinear plots, two or more steps influence the process. Figure 10a–d provides the plots of *q_t_* vs. *t*^0.5^ for Cu(II) ions and phenol sorption onto NaP1 and NaP1CS.

The plots for metal ions and phenol sorption were found to be multimodal with three distinct regions: external diffusion, intraparticle diffusion, and surface adsorption. The initial curved region corresponds to the external surface adsorption in which the adsorbates diffuse through the solution to the external surface of adsorbent. The second stage relates to the gradual adsorption reflecting intraparticle diffusion as the rate controlling step. The final plateau region points out to the surface adsorption and the equilibrium stage, in which the intraparticle diffusion starts to slow down and reaches a plateau. Based on the obtained results it could be inferred that the intraparticle diffusion was involved in the adsorption process, but it was not the only rate-limiting step and that the other steps along with intraparticle diffusion might also be involved. 

As it is shown in Table 4 and Table 5, in most cases the parameters *k_i_* and *C* for Cu(II) ions and phenol on NaP1 and NaP1CS increase along with the initial concentrations of solution (from 25 to 100 mg/L). Therefore, the greater the intercept, the larger the surface sorption trend towards the rate-limiting step [66].

The Elovich model is expressed by Equation (11). This model is used to check whether any chemical reactions occur on the surface [67]. The determination coefficients (*R*^2^) obtained with the use of the Elovich model were lower than those from the pseudo-second order model and comparable to the determination coefficients obtained from the pseudo-first order model [68].

### 3.7. Thermodynamic Study

The thermodynamic studies were conducted at temperatures from 293 to 313 K. The calculated parameters are presented in Table 7. 

The Gibbs free energy change (ΔG°) shows the spontaneity of a chemical reaction. The obtained parameters of ΔG° for phenol and Cu(II) at all temperatures were negative proving that the adsorption process was spontaneous. The value of ΔH° determines whether the process is exothermic or endothermic. The obtained parameters of ΔH° for phenol were negative which shows that the adsorption process was exothermic. However, for the Cu(II) sorption on NaP1 and NaP1CS, the values of ΔH° were positive, suggesting that the process is endothermic. 

The negative value of ΔS° confirms the reduced randomness at the solid–solution interface during sorption of phenol or Cu(II), and the positive value revealed the increase of randomness.

When the reaction process is endothermic, adsorption can increase at higher temperature. This can be attributed to increased surface coverage at higher temperature, expansion, and creation of reactive and active sites. However, when the reaction is exothermic (typical for microporous solid sorbents), a decrease in adsorption at higher temperature is observed. Moreover, the effect of ΔG = ΔH – TΔS is also very important. Typically, entropy decreases because the adsorbent is constrained to two dimensions rather than three (ΔS < 0). Therefore, the expression –TΔS is positive. Thus, the only way for a negative free energy change ΔG < 0 is in the case of ΔH < 0 (the extent of adsorption decreases with increasing temperature). 

The difference between these two system can be explained by the mechanism in which adsorption occurred—in the case of the phenol, the adsorption is physical whereas in the case of Cu(II), it is chemical. Similar results were obtained for phenol, copper ions and nickel ions adsorption onto heat-treated bentonite by Banat et. al. [69], and for phenol adsorption on zeolites by Smart et al. [70] and Yousef et al. [48]. 

### 3.8. Desorption

**Figure 11:**
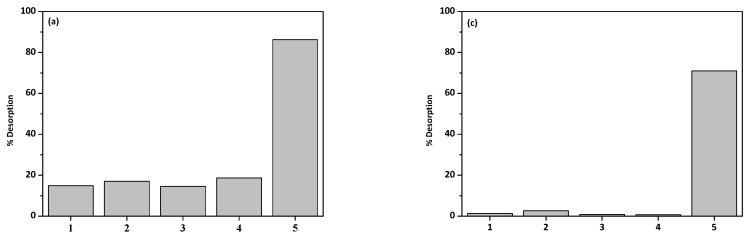

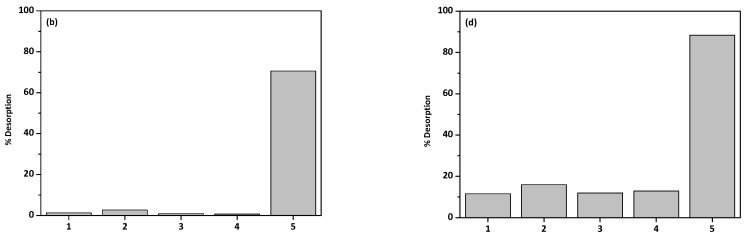
The desorption process of (**a**) Cu(II), (**b**) phenol from NaP1, (**c**) Cu(II), (**d**) phenol from NaP1CS with different desorption solutions 1: 0.5 M NaCl, 2: 0.5 M NaOH, 3: 0.5 M KCl, 4: 30% CH_3_OH, and 5: 0.5 M HCl.

The desorption experiments were performed with the different solutions: 0.5 M NaCl, 0.5 M NaOH, 0.5 M KCl, 30% methanol and 0.5 M HCl. Desorption of phenol on zeolites was also reported by Shah and Mistry [71] and the obtained results showed that the highest desorption rate was obtained with 0.5 M NaOH. Figure 11a–d shows the desorption rate of Cu(II) ions and phenol on NaP1 and NaP1CS. 

For both Cu(II) ions and phenol, 0.5 M HCl was the best desorption factor. The desorption rates for phenol were 86.25% and 88.33% for NaP1 and NaP1CS, and those of Cu(II) ions were 70.56% and 70.99%, respectively. The other desorbing factors reached the desorption rate of phenol at the level up to 18.55% for NaP1 and 15.90% for NaP1CS. For Cu(II) ions it was up to 2.69% for NaP1 and 2.68% for NaP1CS.

## 4. Conclusions

In this paper, zeolite NaP1 and its modified form NaP1CS were employed as adsorbents for the removal of phenol and Cu(II). The following conclusions may be drawn on the basis of the obtained results:

- With the increase of the contact time and initial concentration, the efficiency of Cu(II) and phenol removal increase;
- pH 6 for NaP1CS and pH 7 for NaP1 proved to be the most effective in phenol removal;
- the equilibrium time was determined to be 30 min for Cu(II) and 100 min for phenol on NaP1, whereas the equilibrium time for Cu(II) was determined to be 120 min, and for phenol on NaP1CS, 170 min;
- the Langmuir isotherm proved to be better for the Cu(II) ions, whereas the Freundlich isotherm proved to be better for phenol adsorption;
- the pseudo-second order model near the intraparticle diffusion model proved to be the best one;
- the process of Cu(II) ions sorption was endothermic, whereas for phenol it was exothermic; and
- the desorption study indicated that 0.5 M HCl was the most efficient factor for the removal of Cu(II) ions and phenol from NaP1 and NaP1CS.

Generally, the obtained results indicate that NaP1 and NaP1CS have a great ability for the simultaneous removal of Cu(II) ions and phenol from waters and wastewaters. 

## Figures and Tables

**Table 1:** Textural parameters of NaP1 and NaP1CS before and after adsorption of Cu(II) and phenol.

Sorbent	S_BET_ (m^2^/g)	V_t_ (cm^3^/g)	D_p_ (Å)
NaP1	98.5	0.302	113.8
NaP1CS	53.5	0.134	111.7
NaP1 *	71.7	0.139	87.3
NaP1CS *	56.0	0.123	95.94

* After sorption of Cu(II) and phenol, S_BET_—specific surface area; V_t_—total pore volume; D_p_—the average pore width.

**Table 2:** Phenol adsorption capacity for different absorbents.

Materials	*q_m_* (mg/g)	Reference
Zeolite Y and zeolite Y modified by sylilation	4.24–24.5	[52]
Zeolite X, activated carbon, zeolite X/activated carbon composite	1.4–13.9	[9]
Apatite adsorbents	2–8	[40]
Chitosan and chitosan modified by salicylaldehyde and b-cyclodextrin	1–2.5	[53]
Chitosan-carbon nanocomposite	33.6	[54]
Bentonite	8.43	[55]
Kaolin	2.35	[55]
NaP1 and NaP1CS	4.5–5.0	This study

**Table 3:** Comparison of estimated parameters obtained from the Langmuir, Freundlich, Temkin, and Dubinin–Radushkevich isotherms for Cu(II) on NaP1 and NaP1CS.

Isotherm	Parameters	293 (K)	313 (K)	333 (K)
NaP1				
Langmuir	*K_L_* (L/mg)	0.456	0.283	0.145
	*R_L_*	0.263	0.365	0.528
	*q_e_* (mg/g)	62.45	62.17	64.19
	R^2^	0.984	0.973	0.886
Freundlich	*n*	2.027	1.652	1.153
	*K_F_* (mg/g(L/mg)^1/n^)	15.04	12.48	9.79
	R^2^	0.808	0.796	0.881
Temkin	*A* (L/g)	20.89	0.47	4.49
	*B* (J/mol)	8.53	8.58	11.66
	R^2^	0.931	0.937	0.945
Dubinin-Radushkievich	*X_m_* (mg/g)	239.1	131.2	43.2
	*E* (kJ/mol)	11.7	10.3	8.7
	R^2^	0.828	0.830	0.900
**NaP1CS**				
Langmuir	*K_L_* (L/mg)	0.160	0.152	0.166
	*R_L_*	0.504	0.516	0.495
	*q_e_* (mg/g)	35.35	37.76	37.60
	R^2^	0.976	0.974	0.988
Freundlich	*n*	3.103	2.703	2.293
	*K_F_* (mg/g(L/mg)^1/n^)	7.59	7.01	6.56
	R^2^	0.871	0.793	0.734
Temkin	*k_T_* (L/g)	38.42	15.03	9.92
	*B* (J/mol)	3.52	4.44	4.84
	R^2^	0.949	0.929	0.919
Dubinin-Radushkievich	*X_m_* (mg/g)	1043.3	766.5	540.2
	*E* (kJ/mol)	13.9	12.6	11.5
	R^2^	0.893	0.827	0.779

**Table 4:** Comparison of estimated parameters obtained from the Langmuir, Freundlich, Temkin, and Dubinin–Radushkevich isotherms for phenol on NaP1 and NaP1CS.

Isotherm	Parameters	293 (K)	313 (K)	333 (K)
NaP1				
Langmuir	*K_L_* (L/mg)	0.002	0.001	1.510
	*R_L_*	0.980	0.983	0.051
	*q_e_* (mg/g)	8.40	6.60	4.60
	R^2^	0.629	0.626	0.999
Freundlich	*n*	0.780	0.844	7.169
	*K_F_* (mg/g(L/mg)^1/n^)	228.81	145.46	2.22
	R^2^	0.966	0.903	0.927
Temkin	*A* (L/g)	22.832	20.535	93.076
	*B* (J/mol)	2.17	1.87	0.47
	R^2^	0.683	0.805	0.951
Dubinin-Radushkievich	*X_m_* (mg/g)	806.5	1119.1	1014.5
	*E* (kJ/mol)	5.8	6.1	18.6
	R^2^	0.945	0.872	0.952
**NaP1CS**				
Langmuir	*K_L_* (L/mg)	0.012	0.020	0.004
	*R_L_*	0.868	0.804	0.953
	*q_e_* (mg/g)	14.00	26.00	4.20
	R^2^	0.700	0.848	0.505
Freundlich	*n*	0.560	2.983	0.821
	*K_F_* (mg/g(L/mg)^1/n^)	1204.32	2.67	252.05
	R^2^	0.889	0.939	0.951
Temkin	*k_T_* (L/g)	19.574	1.720	20.485
	*B* (J/mol)	4.02	2.54	1.36
	R^2^	0.900	0.937	0.855
Dubinin-Radushkievich	*X_m_* (mg/g)	85.2	2146.7	1194.1
	*E* (kJ/mol)	4.9	7.9	5.8
	R^2^	0.900	0.938	0.962

**Table 5:** Comparison of kinetic parameters for Cu(II) on NaP1 and NaP1CS.

Kinetics Model	*C*_0_	25 mg/L	50 mg/L	75 mg/L	100 mg/L
NaP1					
PFO	*k*_1_ (1/min)	0.011	0.011	0.011	0.010
	q_e_ (mg/g)	3.95	8.63	12.76	16.35
	R^2^	0.656	0.728	0.705	0.716
PSO	*k*_2_ (g/mg h)	7.479	7.524	6.869	4.866
	*q_e_* (mg/g)	3.95	8.63	12.76	16.35
	R^2^	1.00	1.00	1.00	1.00
IPD	*k_i*1*_* (mg/g min^1/2^)	0.007	0.007	0.013	0.003
	*k_i*2*_* (mg/g min^1/2^)	0.001	0.002	0.001	0.003
	*k_i*3*_* (mg/g min^1/2^)	0.00004	0.00010	0.0006	0.00013
	*C*_1_ (mg/g)	3.920	8.592	12.74	16.31
	*C*_2_ (mg/g)	3.939	8.611	12.75	16.32
	*C*_3_ (mg/g)	3.951	8.625	12.76	16.34
	R^2^_1_	0.9183	0.9189	0.9627	1.00
	R^2^_2_	0.6478	0.7993	0.8176	0.5984
	R^2^_3_	0.7243	0.7251	0.9871	0.7552
Elovich	R^2^	0.814	0.765	0.825	0.804
**NaP1CS**					
PFO	*k*_1_ (1/min)	0.007	0.017	0.016	0.016
	*q_e_* (mg/g)	3.94	8.61	2.58	15.88
	R^2^	0.550	0.652	0.865	0.994
PSO	*k*_2_ (g/mg h)	1.205	0.395	0.089	0.032
	*q_e_* (mg/g)	3.94	8.61	12.58	15.88
	R^2^	1.00	1.00	1.00	0.999
	*k_i*1*_* (mg/g min^1/2^)	18.73	29.24	14.07	8.159
IPD	*k_i*2*_* (mg/g min^1/2^)	0.036	0.215	0.649	0.709
	*k_i*3*_* (mg/g min^1/2^)	0.002	0.112	0.095	0.287
	*C*_1_ (mg/g)	0.001	0.001	0.002	0.027
	*C*_2_ (mg/g)	3.810	7.37	9.54	11.72
	*C*_3_ (mg/g)	3.898	7.97	11.54	12.55
	R^2^_1_	3.921	8.57	12.49	15.04
	R^2^_2_	0.946	1.00	0.875	1.00
	R^2^_3_	0.715	0.647	0.784	0.942
	*k*_1_ (1/min)	0.706	0.921	0.992	0.681
Elovich	R^2^	0.821	0.722	0.858	0.953

**Table 6:** Comparison of kinetic parameters for phenol on NaP1 and NaP1CS.

Kinetics Model	*C*_0_	25 mg/L	50 mg/L	75 mg/L	100 mg/L
NaP1					
PFO	*k*_1_ (1/min)	0.006	0.003	0.005	0.010
	*q_e_* (mg/g)	0.34	0.50	1.16	1.32
	R^2^	0.571	0.918	0.384	0.577
PSO	*k*_2_ (g/mg h)	0.348	0.130	0.088	0.238
	*q_e_* (mg/g)	0.34	0.50	1.16	1.32
	R^2^	0.999	0.998	0.999	0.999
IPD	*k_i*1*_* (mg/g min^1/2^)	0.083	0.027	0.443	0.219
	*k_i*2*_* (mg/g min^1/2^)	0.005	0.006	0.007	0.054
	*k_i*3*_* (mg/g min^1/2^)	0.001	0.005	0.006	0.001
	*C*_1_ (mg/g)	0.027	0.273	0.163	0.581
	*C*_2_ (mg/g)	0.2423	0.321	0.948	0.895
	*C*_3_ (mg/g)	0.2981	0.342	0.979	1.262
	R^2^_1_	0.949	1.00	0.897	1.00
	R^2^_2_	0.904	0.884	0.871	0.707
	R^2^_3_	0.549	0.884	0.979	0.923
Elovich	R^2^	0.845	0.901	0.623	0.820
**NaP1CS**					
PFO	*k*_1_ (1/min)	0.002	0.006	0.002	0.007
	*q_e_* (mg/g)	0.56	0.80	1.32	1.32
	R^2^	0.678	0.740	0.564	0.853
PSO	*k*_2_ (g/mg h)	0.037	0.065	0.030	0.078
	*q_e_* (mg/g)	0.56	0.80	1.32	1.32
	R^2^	0.974	0.997	0.995	0.999
IPD	*k_i*1*_* (mg/g min^1/2^)	0.025	0.029	0.298	0.055
	*k_i*2*_* (mg/g min^1/2^)	0.005	0.037	0.012	0.031
	*k_i*3*_* (mg/g min^1/2^)	0.010	0.006	0.014	0.008
	*C*_1_ (mg/g)	0.191	0.286	0.959	1.00
	*C*_2_ (mg/g)	0.264	0.287	0.938	0.869
	*C*_3_ (mg/g)	0.177	0.569	0.990	0.965
	R^2^_1_	0.805	0.766	0.959	1.00
	R^2^_2_	0.830	0.901	0.938	0.869
	R^2^_3_	0.996	0.997	0.990	0.965
Elovich	R^2^	0.787	0.845	0.954	0.962

**Table 7:** Thermodynamic parameters for the sorption of Cu(II) ions and phenol on NaP1 and NaP1CS.

Thermodynamic Parameter	ΔH (kJ/mol)	ΔS (J/K mol)	ΔG (kJ/mol)		
			293/ K	313/ K	333/ K
**NaP1**					
**Cu(II)**	6.06	24.8	−18.19	−19.32	−21.52
Phenol	−13.08	−76.6	−7.40	–7.22	−6.61
**NaP1CS**					
Cu(II)	2.69	−3.7	−12.98	−14.23	−15.12
Phenol	−26.67	114.3	−8.82	−11.48	−6.21
